# Active inference goes to school: the importance of active learning in the age of large language models

**DOI:** 10.1098/rstb.2023.0148

**Published:** 2024-10-07

**Authors:** Laura Desirèe Di Paolo, Ben White, Avel Guénin-Carlut, Axel Constant, Andy Clark

**Affiliations:** ^1^ Department of Engineering and Informatics, The University of Sussex, Brighton, UK; ^2^ School of Psychology, Children & Technology Lab, The University of Sussex, Falmer (Brighton), UK; ^3^ Department of Philosophy, The University of Sussex, Sussex, UK; ^4^ Department of Philosophy, Macquarie University, Sydney, New South Wales, Australia

**Keywords:** Montessori method, LLMs, learning, 4E: embodied, embedded, enacted, extended, material engagement, active inference and predictive processing

## Abstract

Human learning essentially involves embodied interactions with the material world. But our worlds now include increasing numbers of powerful and (apparently) disembodied generative artificial intelligence (AI). In what follows we ask how best to understand these new (somewhat ‘alien’, because of their disembodied nature) resources and how to incorporate them in our educational practices. We focus on methodologies that encourage exploration and embodied interactions with ‘prepared’ material environments, such as the carefully organized settings of Montessori education. Using the active inference framework, we approach our questions by thinking about human learning as epistemic foraging and prediction error minimization. We end by arguing that generative AI should figure naturally as new elements in prepared learning environments by facilitating sequences of precise prediction error enabling trajectories of self-correction. In these ways, we anticipate new synergies between (apparently) di*s*embodied and (essentially) embodied forms of intelligence.

This article is part of the theme issue ‘Minds in movement: embodied cognition in the age of artificial intelligence’.

*‘Without movement it is not possible to speak of an individual.’* ([1], p. 198)

*‘[M]ental development must be connected with movement and is dependent on it. This is the new idea that* must *enter educational theory and practice.’* ([1], p. 203)

**Maria Montessori, *The Absorbent Mind***

## 1. Introduction

Since the 1990s, cognitive science has increasingly embraced the ‘4E’ paradigm. From work in bio-inspired robotics and approaches emphasizing first-person human experience, 4E understands cognition as embodied, embedded, enactive and extended [2–8]. Recently, the achievements of large language models (LLMs; e.g. ChatGPT) may seem to undermine the 4E claims (e.g. [9,10]). The worry is that, if disembodied systems such as LLMs can generate real understanding through passively inherited ‘second-hand’ information about embodied human–world interactions, then we might doubt the crucial role of embodied and active engagement with the world (e.g. [11–13]). While some have suggested that LLMs are not true cognitive systems and only mimic understanding, here we do not commit to any positions about LLMs’ cognitive status. Although we think that current waves of LLMs do not appear to implement characteristics of true understanding and that some aspects of cognition will likely remain exclusive attributes of embodied and affective interactions with the world, given the speed of LLMs’ development, it is entirely possible that near-future implementations in their generative model or hybrid-robotic systems might change LLMs’ cognitive status (e.g. [14–16]). For us humans, in any case, it seems clear that we attribute meanings to events, knowledge and behaviours by grounding them on dynamic sensorimotor interactions with the world. A more significant and pressing issue concerning LLMs is understanding how cognitive agents like us can best use LLMs’ capabilities, leveraging them in a way that aligns with the human cognitive landscape [17] (see also [18–22]).

One potential cognitive ‘alignment’ problem is that of LLM-mediated learning and education. Given the increasing and ubiquitous adoption of LLMs in various fields and the uncertainty of their future developments, the problem of learning how best to deal with LLMs has become a problem of literacy and therefore of education [23–29].

This article aims to identify characteristics that foster optimal educational environments (specifically schools) for the utilization of LLMs and the enhancement of artificial intelligence (AI) literacy among beings like ourselves, who are both embodied and materially situated. In our analysis, §2 introduces the active inference framework (henceforth AIF), providing the foundational theory for our work due to its comprehensive approach to embodied and enactive learning, with further details in §2b. Subsequently, §3 draws comparisons between the learning processes in LLMs and AIF agents, with a particular emphasis on the role of ‘action’ in AIF learning. Moving to educational contexts, §4 scrutinizes the characteristics of the Montessori method (MM) learning environments. Here, we explore how the AIF sheds light on the neurocognitive achievements of students in MM settings. The central focus of our work is to elucidate how MM classrooms serve as a concrete example of learning environments for AIF agents, as detailed in §4a. We will also illustrate how the MM not only aligns with AIF principles but also resonates with the broader 4E cognitive framework, as outlined in §4b. Hence, the MM exemplifies an embodied and materially situated learning environment, embodying the kind of ‘clear educational approach’ that Kasneci *et al*. ([30], p. 1) deem essential for the successful integration of LLMs into classroom settings.

## 2. The active inference framework

### (a) The basics

Active inference is a theory of action and perception based on the free energy principle (FEP) [31,32]. The FEP states that any self-organizing system separated from its environment by a boundary that persists through time must, by definition, preferentially occupy a limited number of ‘expected’ states that are the most probable states given its phenotype (e.g. human temperature is probably 37°C). The AIF aims to deliver a process theory explaining how agents remain within expected bounds, minimizing uncertainty within volatile environments [33,34]. In AIF terms, the brain encodes a probabilistic generative model of the causes of its sensations, which it leverages to generate action that move it away from deleterious exchanges with the environment, bringing the system closer to those expected states conducive to survival. This, in turn, requires a method of efficient learning. Rather than passively waiting for incoming sensory information, systems are proactive, generating ‘top–down’ predictions about future states [33,35]. Predictions, produced by expectations encoded within the generative model, underpin perception and action. If predictions fail to match the incoming sensory data, ‘prediction errors’ are generated that propagate upward through the hierarchy of the generative model, driving action and revised prediction. Not all predictions are of equal importance or reliability; a mechanism known as ‘precision weighting’ modulates the impact within the overall system that sets of errors have: high weighting drives learning and further engagement, while low weighting renders error units relatively impotent [33,36]. Precision also applies to action, representing the degree of confidence that the system has in an action to reduce uncertainty, central for adaptation.

Prediction errors can be minimized in two ways. First, the system can update the generative model to better fit incoming sensory data, a process known as ‘perceptual inference’ [33,37]. Second, the agent can engage in actions to elicit an expected state that, on some accounts, are strategies of action readiness (e.g. [38]). Revised (more accurate) predictions better ground the agent to act effectively: the embodied brain, is, however, as interested in changing the world to fit its own predictions as it is in describing how things currently are [38]. Parameters dictating which states are ‘surprising’ depend on organisms’ developmental and evolutionary history. For human agents, these expectations are complex and sometimes culturally informed. For example, an expectation that body temperature will be around 37°C is essentially ‘hardwired’: basic metabolic processes (e.g. shivering or sweating and associated sensations) drive behaviour that maintains body temperature. However, the specific behaviours that an agent will take in any given situation are embedded within its cultural and social context: depending on the situation, we could find it more appropriate to sit and shiver in silence or to request a blanket.

### (b) Active inference is enactive and embodied

There is much debate concerning the extent to which active inference is (or fails to be) compatible with the wide range of claims represented under the umbrella of 4E cognition. Much of this debate revolves around the extent to which reliance upon internal generative models suggests (or fails to suggest) a kind of ‘veil of representation’ separating the thinking being from the world: a veil that is—some theorists argue—quite against the spirit of the 4E revolution (e.g. [39]). Others (e.g. [33]) argue that a strong antipathy towards internal models and internal representations ought not be part of the 4E package in the first place. According to Clark, active inference suggests modes of representation that are profoundly ‘action-oriented’ (geared to selecting actions that themselves do much of the apparently ‘cognitive’ work) and in this way provides an ideal platform for understanding the role of the brain in orchestrating embodied action. For present purposes, we do not need to take a stance within this complex landscape (arguments about the role of rich internal representation versus more sparse, embodied interpretations of the framework). Instead (in line with Ciaunica *et al*. [40]), we highlight the power of the AIF to provide a principled account of action itself. That account depicts action as a key means of minimizing long-term prediction error, either by changing the world to fit the predictions (standard goal-directed action) or by sampling the world in ways designed to increase our states of information (‘epistemic action’). We return to this crucial notion, placing it in the context of active inference and education, in §4.

In the remainder of this section, we support our (highly action-oriented) interpretation with work in active inference that highlights the deeply embodied nature of action and action selection. Perhaps the most striking example is the part of the framework known as ‘error dynamics’. Despite the framework’s emphasis on the minimization of uncertainty and prediction error, error dynamics highlights the way that error can be inherently valuable. In short, some forms of uncertainty signal that there is learning to be done and that there’s an opportunity to improve our predictive grip on a given situation—often by means of some form of active engagement with the world. For example, a fan of Sudoku will gravitate towards puzzles that are not too easy but not impossibly hard either. These are the ones where a certain action (writing down a guessed number) can sometimes resolve more than expected amounts of persisting prediction error. We will tend to occupy ‘learning sweet spot’ spaces like that until they are mastered, at which time we increase the challenge. Attention to error dynamics of this sort helps explain why it is that agents are sometimes driven to both curate and resolve prediction errors. This, in turn, has been linked to the affective responses that (it is hypothesized) are a kind of agentive indicator of the sweetness—or otherwise—of the current learning situation. It quite literally feels good to be resolving errors at a greater-than-expected rate. In other words, agents don’t seek only to minimize prediction error in the present but are sensitive to rates of change in prediction error minimization over time, relative to their prior expectations about that rate, i.e. whether they are reducing error better than, or slower than expected [41–44]. The sense of rising frustration we feel while sitting in an unexpected traffic jam reflects us minimizing error (relative to the action-guiding prediction of arriving soon at our destination) more slowly than expected (e.g. [45]; see also [46–48]).

These affective states aren’t a mere by-product, though, but instead are a crucial feature of action selection. Unexpected increases or decreases alter the precision weighting on action policies [44,49]. Doing better than expected makes agents more confident in their actions (increased precision weighting). But when expectations are not met, they downregulate the precision of those policies. In this way, error dynamics play a central role in keeping agents tuned to the actions that work and lead us to try new strategies when actions fail to bring about even the expected rates of error minimization. This is important because even the best action policies can have an expiry date: rich foraging areas, for example, will only provide food for so long. Thus, error dynamics drive exploration and implement a form of curiosity. They also mandate play (which we return to later in more detail). Research has shown that because agents find pleasure in reducing manageable uncertainty, they are attracted to just-uncertain-enough environments: the ones that offer such opportunities (see, for example, [50–53]).

## 3. Learning in LLMs and active inference agents

AIF learning can be cast as the development of generative models that enable the realization of expectations through sensorimotor engagement with the environment [34,54]. Sensorimotor engagements maintain control over important physiological setpoints and, when combined with affordances that are opportunities for highly expected actions (e.g. eating and drinking), become meaningful for the agent. In this sense, AIF learning is highly active. The agent is the locus of meaningful engagements between itself and the environment, driving the development of its own deep, causal generative models, which, in turn, actively alters the dynamics of its own engagement with the environment (e.g. [55–57]).

Contrariwise, the learning of current LLMs is passive. The generative model (a deep neural network), exposed to large quantities of data, adopts rules based on either back-propagation or evolutionary algorithms to explore the parameter space and minimize error (i.e. deviation to the expected function). Back-propagation means computing local variations of errors given variations in individual connection strength: adjusting the strength in order to minimize error back-propagates the error signal [58]. Evolutionary algorithms compute a family of solutions that averages their parameters, with each solution having a weight inversely proportional to its error [59]. Both require an abundance of computing power and give no guarantee that the optimal solution within a dataset means learning relevant structures: a system recognizing a certain input (for instance, images of cats) within a given dataset may fail to recognize the same inputs in a different dataset. AI systems, including LLMs, exploit this basic model of optimizing many parameters at great computational costs and with little oversight. Commercial generative AI systems based on transformer architectures [60] use a very simple ‘self-attention’ mechanism enabling them to efficiently retrieve contextual information. While other deep learning systems function by applying successive filters for inferring more abstracted features, LLMs add a generative aspect: they ‘learn’ by predicting the structure of the data and are automatically optimized to fit the observed data points. If a generative AI is trained to recognize cats, it generates a space of cat pictures (a probability distribution over the category is treating) and compares them against the picture under examination. This is the reason why LLMs generate convincing images or word streams: the reproduction of data points is in its core architecture.

In the case of ChatGPT, the training of the model occurs offline,^1^ on human-produced textual data taken from the internet. The model is provided with input–output pairs: texts (or other inputs; e.g. [62–64]) from which part of the information is missed and of which is asked to predict the missing part (output). When predictions are accurate, the model is ‘rewarded’ (its accuracy scored through an assigned number) and then fine-tuned with an increasing quantity of texts, missing larger portions. Finally, performances are evaluated, validated and tested on datasets to ensure the model generalizes accurately to unseen data. Through different degrees of supervision, the network is optimized for effectively predicting the most likely output, based on associations and contrasts between inputs and data in its training set. That is, the model can predict, with a high level of accuracy, the portion of the missed text, which corresponds to answering the question asked by a user. LLMs are powerful tools: users leverage the (approximately) 2.25 billion pages contained in the indexed web and, through ‘prompt engineering’, craft their prompts in targeted ways to exact useful responses. The capabilities of LLMs have already been applied to a wide range of cases, such as the ability to decode the meaning of perceived and imagined speech and silent video [65]. Results are impressive, but they also highlight inherent limitations, particularly the flexibility in transferring knowledge from one set of data to another, which also bring about states of ‘artificial hallucinations’ (i.e. generated responses that are either factually incorrect, non-sensical or not grounded in reality (e.g. [66–68]).

Current LLM systems do not implement forms of active learning and autonomous engagement with the world’s affordances. Even if the system could be able to recover facts about the world’s structure, the recovered facts are explicitly based on the 4E human experience, hence the depiction of LLMs as ‘stochastic parrots’, due to their pattern-matching approach to the language detailed above [69].^2^ Others consider LLMs only an outward display of intelligence: similarly to Searle’s ‘Chinese room’, they give the impression of comprehending only because they can match incoming input symbols with specific output symbols [74]. Obviously, it is not possible to say that disembodied entities such as LLMs could never achieve understanding or agency in some sense. The production of systems able to describe the reasons for their decisions (i.e. explainability) in natural language, as a pragmatic premise to overt discourse, is a major avenue for AIs’ development [14–16]. However, even if LLMs could be considered bona fide cognitive agents, this (as we noted earlier) does very little to undermine the position of 4E and AIF accounts of cognition. On the one hand, the ‘ground’ of LLMs’ understanding will be explicitly based on the 4E human experience (e.g. [75]). On the other hand, despite the principled similarity between the generative architecture of LLMs and AIF agents, AIF models are always primarily concerned with how the agent is able to act, reducing prediction errors over an integrated array of interoceptive, proprioceptive and exteroceptive sensations, and grounding their understanding of language in their biological, social and cultural activity [76]. Embodied and in the business of active prediction error minimization, AIF agents aim to understand the underlying dynamics of the agent–environment coupling and model themselves as the ‘cause’ of changes to their own sensory states, developing their own sense of agency through active engagement and intervention onto the world (e.g. [55]).

## 4. Active inference goes to school

In the previous section, we described the process of learning in LLMs as passive, mainly resulting from a training process or from implementations in their generative model. In this section, based on the conception of learning of the AIF framework, we look at how learning occurs in embodied human agents such as us. We consider learning as being driven by active engagement with the environment. We then discuss the effect of LLMs on learning in embodied agents, within the context of the classroom.

In AIF terms, development (i.e. the construction of cognitive competences in relation to the physical and biological growth of the body) and learning (i.e. the refinement of cognitive competences and the acquisition of knowledge), cognition, skills and understanding are all result of the same process of epistemic foraging, precision weighting and prediction error that an AIF agent starts as a foetus in the womb, interacting with its own ‘learning space’—the mother’s uterus [40,77] (see also [78,79]). Sensory feedback, minimal knowledge and competences constructed as results of the foetus’ own activities allow the infant to be born with strong expectations about being the cause of changes in its own sensory inputs and in the environment. For instance, if—as a foetus—it kicks when hungry ‘summoning’ the mother to eat (e.g. 80), as an infant it cries, expecting to obtain food. Crying is a highly precise action policy: the infant learns that its own crying causes the parents’ various attempts to minimize its uncertainty along a wide range of responses [76]. The role of undertaking autonomous activities—even random as infants’ ‘motor babbling’ (i.e. the flailing of the limbs [81,82]; see also [83–85])—for the development of skilful capabilities (i.e. goal-directed actions), has been shown by work in embodied robotics adopting the AIF. Research on social understanding has instead shown the importance of engaging with the material world. For instance, 3-month-old infants can neither proficiently grab objects nor understand goal-directed actions performed by others. However, once given the opportunity to manipulate toys through little Velcro-mittens, they learn to recognize identical actions performed by an experimenter wearing a similar apparatus [86] (see also [87,88]).

In AIF terms, active intervention and engagement are not limited to behaviours such as grasping and kicking but also apply to perception and sensory predictions, which are adopted for sampling the environment and learning from unexpected states. The infant, an ‘active seeker’ ([89], p. 148), makes use of its visual perception for epistemic foraging, as implicitly acknowledged by the adoption of the violation of expectation (VoE) paradigm in studies on infants’ cognition. The VoE relies on the eye-tracking measures of the amount of time that an infant spends observing a certain event; the longer an event is looked at, the more it is ‘surprising’ or unexpected for the child [90–96]. In AIF terms, this ‘surprise’ signals information gain; unexpected events are a prediction error from which the infant learns through active observation. Applied to educational settings such as schools, this translates into a careful attention to the embedding experience of the learners through the design of the materiality of learning (i.e. spaces and educational materials).

We chose to focus on the MM because it is based on the idea that development and learning are in fact the same process, underpinned by the active engagement of the pupils with their learning space.^3^ It describes an applied educational system aiming at ‘optimal development’, rather than academic success as traditionally understood (e.g. [97–100]; see also [101,102]). While the MM theoretically insists on the materiality of learning as enabling and aiding cognitive functions, as other theories of children’s development and learning (e.g. [101–106]), it is an applied ‘formal’ educational setting, essentially organized around an indirect form of teaching, in which pupils develop and learn dynamically by repeatedly interacting with their learning spaces and materials. Compared to today mainstream educational science that ‘has only recently started to pay due attention to the significance of the material and spatial dimensions of learning’ ([107, p. 325]; see also [108]), the MM has a long-standing tradition of so doing and has been deployed in a wide variety of cultural and social contexts (e.g. [109]; see also [110]).

### (a) Active inference learning in a Montessori classroom

In terms of schools, the most traditional and well-known approach (at least in western societies) is surely the ‘teacher-centred’ approach in which teachers supply the knowledge that pupils must acquire and then evaluate the efficiency of the training through tests, grades, etc. (e.g. [111]; see also [112–114]). Despite being necessarily multimodal (e.g. books combine images and written text, or music classes, sounds and written notes; passing a test has social and emotional components), these ‘traditional teacher-centred’ methods bear similarities to the training of disembodied entities such as LLMs. Pupils are trained on increasingly complex material produced by humans (a curriculum) and asked to generate texts or answer questions such that the teacher can evaluate if pupils can correctly retrieve the information. These methods rely exclusively on teachers’ ability and interest in delivering, adapting and evaluating information (e.g. [115]). That is, they exhibit unidirectional and linear transmission of knowledge from teacher to student, where the teacher has an active role and the student is a passive vessel to be filled [116] (see also [111,117–119]).

Different, less ‘traditional’ educational theories, sharing the common belief that actively engaging with educational artefacts and learning ecologies allows pupils to construct their own knowledge and understanding, often eschew extrinsic evaluation systems like grades and exams and focus more on personal interests and curious exploration (e.g. constructionism [120–122]; democratic school [93,123]; Reggio Emilia approach [124]; place-based education [125]; learning by design [126,127]). Among them, we chose to focus on the MM, conceived by the Italian physician Maria Montessori at the beginning of the twentieth century, because it is ‘organized to the core’ ([128], p. 19) around the idea that development and learning are embodied and enacted processes, spontaneously unfolding through the dynamic interaction between children’s autonomous exploration and their material embeddings.^4^ Recognizing that ‘cognizing is a matter of embodied engagement’ [132], that sensorimotor system and cognition are intertwined and that knowledge, concepts and higher cognitive functions are brought about by sensory and motor activities, education occurs through the design and organization of the materiality of learning (e.g. [89,133,134]; see also [135,136]). Educational materials and physical classrooms are designed by Montessori herself as affordances for perceptual and active exploration and for driving attention [89,100,128,136–143]. The MM can be structured around four core ideas that, might be worth noting here, are the same and with roughly the same meaning as the related adopted by the AIF: intrinsic motivation, attention, precision and error control [139,144,145]. At the bottom, there is the intrinsic motivation of the child to act, exploring and freely choosing tasks. Never driven by external rewards such as grades, children develop, learn and achieve understanding, self-determination and autonomy because of their own enacted exploration and embodied intervention in the world [89,128,139,146–148] (see also [149]). If obstructed in their movement and in their choices of activities, children fail to fully develop intellect and personality, ‘incapable of controlling the way they interact with their circumstances’ ([138], p. 39). Chosen activities are meaningful, and the child focuses its attention and performs them with an autonomously established increasing precision [150]: ‘whether moving blocks or … composing poetry, agents engage in activities with internal normative standards that allow them to do the activities well’ ([140], p. 49; emphasis in the original). Precision is obtained overcoming mistakes: children need to cultivate ‘a friendly feeling toward errors’ and not be scared ‘to knock into things’ (Montessori, in Frierson [140], p. 50). ‘A “control of error” that allows students to recognize manageable goals and correct errors’ ([140], p. 50) is then implemented in the materials and in the space. Free to explore, pupils form their own cognition (‘mental meat’, in Montessori [89], p. 67) through their dynamic interaction with the environment. Therefore, they are taught indirectly, through their material engagement with their learning space, the classroom. Known as ‘prepared environment’, the classroom is an immersive ‘ecology’ of purposefully selected and organized artefacts that allows students of different age and developmental stages (classes are age mixed, usually first to third or first to sixth grade, and so on) to extrapolate knowledge from everyday activities and multiple times at different levels of development [133,146,150]. Furniture, objects and materials, all of the correct size and weight such that children can effectively interact with them (e.g. [128,134,140,151,152]), are purposedly organized in the space tailoring interests and developmental trajectory of each child [153–157]. Artefacts, classrooms and the whole curriculum are designed to help pupils construct abstract knowledge and are structured around the idea that minds develop through active intervention onto the world (e.g. for mathematics, see [142]).

For instance, the study of ‘natural sciences’ such as botany is organized around the ‘observation of nature’ (e.g. outdoor exploration in or around the woods and seasonal nature-related activities) and gardening (e.g. [158–161]). Natural elements collected during these activities (e.g. leaves, branches and plants) become a physical part of the classroom and multisensory educational materials. While plants can be cared for as part of the ‘exercises of practical life’, leaves and pieces of wood can then be compared to the images on the ‘botany cards’ (i.e. cards with plants’ picture, biological classification, Latin nomenclature and secular name) or to the geometrical shapes of the ‘botany cabinet’ (i.e. sets of carefully geometrically designed wooden leaves/inserts), or used for artistic productions (for instance, as ‘stamps’), according to desires and interests of each child. Since MM’s curricula span 3 years (not just 1 year or a few months), the child will encounter these activities multiple times and at different stages of development, allowing the construction of abstract concepts (e.g. ‘life cycles’ and ‘ecosystems’). On the other hand, the interaction with these natural elements within the ‘prepared environment’, for instance, displayed by the teachers within or upon the shelf of ‘science’ in physical proximity with books on related topics, allow the children to learn (i.e. construct) abstract associations between everyday activities and abstract knowledge (e.g. gardening and natural sciences) and across domains of knowledge (e.g. art, geometry and science) [128,162–164]. In this way, the children not only develop ownership of their learning space, modified through their own intervention, but also of their own knowledge, meaningful because constructed through their own free exploration (e.g. [128]).

Empirical studies have found that Montessori-schooled children (compared to traditionally-schooled) seem to perform better across a range of measures (e.g. [100,128,139,141,165–169]) and that Montessori schools displayed smaller disparity in performance between children from low and high income and from diverse cultural backgrounds [128,150,170,171]. Adopting the AIF, the relative success of the MM can be explained primarily by focusing on the importance of exploration, curiosity and playful behaviour in learning. Some theorists claim that play doesn’t feature in the MM: ‘play is called “work” … in the sense of “play is the child’s work”’ [150, p. 1198]; there is no room for fantasy or make believe (e.g. [128,172,173]) and ‘children play with hands-on materials in order to learn academic content’ [150, p. 1197] (see also [174,175]). While it is generally difficult to delineate play from non-play (e.g. [176]), the whole Montessori curriculum when interpreted through the lens of the AIF is unequivocally playful. Such tasks—low stakes, controlled and engineered—allow children to explore their capabilities against relatively surprising or challenging situations. According to the AIF, actions like play and curious exploration, albeit metabolically costly, open a space of greater possibilities for effective (epistemic) actions that minimize the prediction error over longer timescales, collapsing the distinction between exploitative and explorative behaviours [177]. As mentioned earlier, changes in the rate of error reduction are linked to bodily affect. What we have then is a deeply embodied and affective drive to explore the edges of our capabilities. We seek out uncertainty as a possibility for better-than-expected slopes of prediction error reduction, where short-term spikes in prediction error can lead to longer term downward trends in uncertainty: a child, while soaking itself and the floor playing with faucets in the bathroom, understands the causal regularities that underpin skilled use of faucets [177]. Additionally, for play being play, the agents need to be outside of the pressures of competition and survival, deliberately seeking out scenarios that provide resolvable uncertainty. According to AIF, play requires a ‘context of freedom’ ([177], p. 463) and goes beyond ‘make believe’ and fantasy. In this sense, the MM is one that truly discovers the inherent link between play and learning suggested by the AIF and that features heavily in many theories of play (e.g. [178–180]).^5^ Leveraging exploratory behaviours, facilitated by ‘manufactured’ manageable prediction errors, to allow an agent to engage in the resolution of uncertainty at the edge of its capabilities, play is then a form of epistemic foraging. Understanding play as epistemic foraging explains why children are so attracted to novel stimuli (objects expected to offer the most resolvable uncertainty) [181] and why young children, especially those in Montessori schools, don’t seem to have the same starkly negative affective responses to errors that adults do [182].

Montessori classrooms, thought of as crafted epistemic niches made up of affordances that aim to keep children on the edge of their capabilities and truly allow students to ‘experience first, signify later’ [132], offer opportunities to make errors. Materials and designs perceptively enhance errors, so that children can perceive and detect mistakes by themselves and might be stimulated to undertake self-corrective actions [128,183–186]. In this sense, we can say that educational materials and prepared environments are designed as ‘modes of skilful engagement’ ([187], p. 45), such that they maximize the precision weighting of any resultant prediction error signals. For instance, in a maths exercise, children encounter a spindle box, a box made of wood and divided into 10 compartments from 0 to 9 and having 45 spindles (1 + 2 + 3 + …. = 45). The child notices to have made a mistake when there are not enough spindles for correctly filling the remaining compartments [128]. Then, re-positioning the spindles in the box, the child acts to confirm the original belief that all the spindles have a certain accommodation, understanding where and when the mistake has been made.

Neurocognitive studies confirm that Montessori-schooled children are particularly good at error monitoring (i.e. ‘the intrinsic ability to detect and evaluate outcomes that violate expectation and to adapt in response’ [188]). Encouraged to freely explore and to make and monitor their own mistakes, Montessori-schooled children also exhibit a distinctive affective response to errors, processing their errors and successes comparatively neutral—whereas children who undergo traditional-schools’ testing and grading in terms of success or failure show strong aversive responses to mistakes [182]. This disparity can be explained by adopting the AIF, which understands affective states as reflecting changes in the rate of error minimization relative to expectation [42,189]. Engaging with affordances in their epistemic niche, agents not only update expectations regarding features of the environment but also about their own capacity to engage effectively [141,190]. Montessori environments afford not only children’s epistemic foraging but also the fine-tuning and updating of their expectations about what happens when they face uncertainty. For a Montessori pupil, mistakes aren’t strong VoEs. They are, rather, part of the ongoing, highly active process of experimentation and exploration.

### (b) LLMs and the future of the classroom

In the previous sections, we have suggested an approach to learning within educational settings that targets embodied and enactive capabilities using designed learning environments. Other work exploring 4E approaches to education includes that of Kraus & Wulf [191], Macrine & Fugate [192], Hutto & Abrahamson [132], Videla *et al*. [193], Nguyen & Larson [194], Pouw *et al*. [195], Kiefer & Trumpp [196] and Mathias & von Kriegstein [197]. Very much in line with our picture, Hutto *et al.* [198] write that: ‘You cannot directly teach anyone anything; at best, you can create activities that foster opportunities for a person to construct some targeted knowledge for themselves’ (p. 377). Our focus on the MM is, in a sense, merely pragmatic: the MM is an educational philosophy plenty available and applied in schools (e.g. [109,199]). It is already grounded on the idea that ‘Education is not what the teacher gives, but a natural process that happens within the human individual ... The task of the teacher … is that of preparing and arrange a series of motives of cultural activities in a specially prepared environment’ ([89], p. 47). MM’s classrooms and artefacts are already opportunities for material engagement (*sensu* [200]) and—particularly true for mathematics—are already designed ‘making room for the possibility that nonsymbolic, nonconceptual embodied activity is constitutive … of certain kind of competence or knowledge’ [132], accommodating ‘nonconceptual, nonsymbolic and symbolic’ cognitive artefacts [132] (e.g. [100,142,201–205]). Looking a little further afield, but still within the 4E family of work, the MM nicely reflects the idea from cognitive archaeology that ‘thinking is thinging’ [187]. Classrooms and educational materials are not simply external tools, separated from the agent’s cognitive functions, but instead ‘modes of skilful material engagement’ ([187], p. 45), in which ‘hands and tools are made for action in action’ ([187], p. 48; for a general discussion about tools, see also [206,207–210,211]). It might not be a coincidence, then, that Montessori (like Malafouris [187]) dedicates extensive attention to the relationship between the mind, the hand and its movement: ‘When man thinks, he thinks and acts with the hands … The development of the hand’s ability goes at the same speed as the development of the intellect’ ([89, p. 204]; see also [212–216]). Insisting on the materiality of learning and considering cognitive functions as established through movement and exploration, the MM is compatible with a variety of 4E approaches. The distinctive contribution of the AIF is to show, in rich formal detail, with established mappings onto brain structure and function, exactly how it is that these cognitively important environments and interactions do their work. What the AIF gives us is a detailed framework for interpreting these effects in terms of the minimization of prediction errors by action, the tuning of precision, the role of affect and interoceptive feedback and the automatic shifting between exploit/explore dynamics (e.g. [217,218]).

Manufactured learning environments that leverage exploratory behaviours and facilitate manageable prediction errors are then fundamental when we turn to imagine the implementation of different, by definition, disembodied artefacts such as LLMs. LLMs’ potential impact on education has generated a variety of responses, from fear to enthusiasm to immediate applicative attempts in a variety of contexts (e.g.[219–225]; for a summarizing commentary, see [30]). If it seems clear that LLMs are here to stay and their integration in education is not challenge-free, then ‘a clear pedagogical approach with a strong focus on critical thinking and strategies for fact checking are required’ ([30], p. 1). That is, it isn’t enough to simply have LLMs lying around, using them to produce and spoon-feed information on command. Their functions, strengths and limitations need to be overseen in a structured way, since—as disembodied entities—LLMs ‘are fundamentally incapable of understanding people’s embodied interactions’ [226]. While it is beyond the scope of the space remaining to really do justice to the question of how LLMs might, or should, feature in classrooms, it’s worth signposting some considerations about their potential adoption as part of the ‘prepared environment’ and linked to the irreplaceable role of the teacher. Teachers, specifically within the MM, act as mentors: they organize, watch over, communicate and (most importantly) collaborate with pupils in their exploratory behaviours, intervening as little as necessary (e.g. [227–231]).

According to the motto of Montessori’s schools: ‘Help me in doing by myself’, teachers do not correct, or do for the children, but accompany them in learning how to correct their own mistakes, relying on embodied capabilities and personal interests [104]. With this in mind, LLMs could be powerful educative ‘tools’ (*sensu* [187]) in the hands of teachers, augmenting the growing skilled capabilities of children (e.g. [232–234]). Here’s an example of how this might look: as a child is engaged in a writing task, rather than making autocorrections, the system could highlight mistakes, draw the child’s attention and suggest helpful resources for self-correction [235]. In this way, LLMs, as material cognitive prosthetics, could be adopted to ‘provide new interactive possibilities for moving forward to learn new [skills]’ ([187], p. 50).

## 5. Conclusions

In this article, we have suggested that it may be fruitful to approach education as a process whose aim is to nudge embodied interactive agents along empowering dynamics of prediction error minimization. Understanding education in this way also revealed, at least in outline, some of the ways essentially embodied, active beings such as ourselves might learn to make better use of new resources such as those of generative AI. To this end, we have adopted the AIF (§2), a theory of cognition deeply embodied and embedded that interprets learning as result of the dynamics of error minimization and precision weighting of sensorimotor predictions. In this work, we claim that, albeit human cognition and the core architecture of LLMs are both formalized in terms of generative models (§3), the resulting learning is different. LLMs seems to learn passively, as a result of their training, while learning in human AIF agents, particularly children, happens because they actively engage in multisensory and exploratory policies. Therefore, in §4, we firstly describe learning as the generation of increasingly precise sensorimotor activities that, in human AIF agents, start early, already in the womb. These same activities allow learning in formal educative contexts such as schools. For this analysis, we introduce the MM educative system (in §4b) that we choose because, similarly to the AIF, consider cognition and sensorimotor activities intertwined. Additionally, the MM stresses the role of the materiality of the learning (materials and classrooms) as affording exploratory behaviours and manufacturing solvable uncertainty. In §4b, we introduce other 4E account of education, which we find quite in line with our interpretation of the MM in AIF terms. Finally, we suggest that LLMs could be adopted as powerful cognitive prosthetics, able to afford self-corrective and explorative behaviours within a structured learning embedding such as the ‘prepared environment’ of the Montessori classroom.

## Data Availability

This article has no additional data.

## Footnotes

^1^
The case of online training of LLMs has been explored by Microsoft’s Tay, which was left to learn from online Twitter interactions and was, somewhat unsurprisingly, very quickly coaxed into parroting misogynist and racist nonsense [61].

^2^
Although this might be unprecise and maybe unfair to parrots who are quite able to autonomously engage with world affordances (e.g. [70,71,72]). For a general account, see [73].

^3^
Also in Montessori, as in the AIF, the cognitive life starts early, in the womb, to which she refers to as ‘psycho-embryonal life’ (e.g. [89], p. 111).

^4^
The similarity of these ideas with the well-known ‘genetic epistemology’ of Jean Piaget, which some readers might notice, should not surprise. Piaget had done his first observations in a Montessori school and attended at least one of Maria Montessori’s classes for educators [128, p. 27, 129,103]. Most importantly, Piaget was for many years the president of the Swiss Montessori Society (e.g. [130]). It is worth noticing, however, that while Piaget’s theory of development is a series of discrete stages, for Montessori development is a continuous, in which ‘each level descends from the previous one and in turn prepares the next’ ([131], p. 67).

^5^
It also elegantly accounts for the positive affect associated with playful behaviour. On this account, play feels good because any change in the rate of prediction error over time relative to the agent’s expectations manifests in the agent’s phenomenology as bodily affect. This deep linkage between learning and feeling is potentially very helpful for elucidating and weakening the apparent tension between learning (as work) and play (e.g. [150]).
